# The fiction of health Services

**Published:** 2012-06-30

**Authors:** Oscar Echeverry

**Affiliations:** Senior Public Health Specialist (retired), World Bank.

**Keywords:** Health services, medical services, health protection service, health promotion services, preventive medicine services

## Abstract

What we know today as Health Services is a fiction, perhaps shaped involuntarily, but with deep health repercussions, more negative than positive. About 24 centuries ago, Asclepius, god of medicine, and Hygeia, goddess of hygiene and health, generated a dichotomy between disease and health that remains with us until today. The confusing substitution of Health Services with Medical Services began toward the end of the XIX century. But it was in 1948 when the so called English National Health Service became a landmark in the world with its model being adopted by many countries with resulting distortion of the true meaning of Health Services. The consequences of this fiction have been ominous. It is necessary to call things by their names and not deceive society. To correct the serious imbalance between Medical Services and Health Services, Hygeia and Asclepius must become a brother and sisterhood.

## Abstract (es)

Lo que hoy conocemos como servicios de salud es una ficción creada, talvez involuntariamente, con hondas repercusiones, más negativas que positivas para la salud. Hace unos 24 siglos, Esculapio dios de la medicina e Hygeia diosa de la higiene y la salud generaron una dicotomía entre enfermedad y salud que se mantiene hasta hoy. La substitución de los servicios de salud con servicios médicos comenzó confusamente a fines del siglo XIX. Pero fue en 1948 cuando el llamado Servicio Nacional de Salud inglés marcó un hito en el mundo y su modelo fue adoptado por muchos países, tergiversando el sentido de los verdaderos servicios de salud. Las consecuencias de esta ficción han sido nefastas. Es necesario llamar las cosas por su nombre para no engañar a la sociedad y corregir el grave desequilibrio entre los servicios de salud y los servicios médicos. Hygeia y Esculapio deben hermanarse.

## Introduction

What we know today as health care is a fiction created perhaps unintentionally, but it has had a profound impact on health, more negative than positive. The concept of health has been studied, for now let's stay with what everyone feels like is most appropriate, still its complexity does not allow us to herein attempt a judicious analysis that can add clarity to the central issue presented in this essay. Yes it is worthwhile to say that since the days of Asclepius, god of medicine, and his daughter Hygeia goddess of hygiene and health, a dichotomy has been established in the thinking on disease and health that has remained for over 24 centuries^1^. At first, Asclepius was worshiped for his ability to ward off disease and restore health, while Hygeia slowly faded with her teaching on how to preserve and maintain health through hygiene practices and compliance with the laws of nature.

Only after about 18 centuries when Asclepius was not able to contain the ever more frequent pests in the world that there was a resurgence of Hygeia demonstrating that she was able to control epidemics and thus giving great prestige to the field of public health. At the same time a confusing idea began to spread that was to call health services to the attention of patients, organized mainly by insurance companies and the State*. Using the fruits and prestige achieved by Hygeia in health promotion and disease prevention, Aesculapius began to call health services his medical services.

*(Only some organizations of civil society in Europe created "sickness funds" to finance medical care for its associates).

### The fiction

The event that marked the decisive turning point of this fiction occurred in 1942 when William Beveridge introduced into the English Parliament a Plan for Social Security and Allied Services, which included an English Department of Health which in article XI described it as follows: "The medical treatment that covers all requirements will be provided to all citizens by a National Health Service organized in the health departments ..."^2^. Only in 1946 did the English Parliament approve the first National Health Service and began its organization and operation in 1948. It was based on three core principles relating to the individual and not to the general population: "1) meet the needs of every individual; 2) based on clinical need, not on ability to pay and 3) be free at the point of entry"^3^.

Therefore, it provided for a tripartite structure based on hospitals, family doctors, dentists, optometrists and pharmacists, and community clinics to provide immunizations, maternal care and medical services to students. That is, the system was limited to supporting the practice of individual medicine, to cure, to control, to mitigate and in some cases to prevent the disease. For Beveridge, a fundamental argument - in times of war - was that it would dispel the fear of illness for the citizen by removing financial barriers that impeded access to medical and hospital care.

The same British Medical Association (BMA) had formulated its own National Health Insurance Plan in 1938 but more as a mechanism to protect MD´s from patients unable to pay than to secure these patients against the high costs of medical care^4^.

The proposed medical plans were relegated to oblivion with the new health care, to the point that in 1945, an English doctor harshly criticized the scheme proposed by the government warning of the privilege of Aesculapius over Hygeia: "Much has been heard of professional reactions of doctors to the scheme, including their financial status, but little has been published to show the fundamental flaws of the scheme that legislates only for disease and fails to initiate a creative health policy"^5^.

Many countries in the world followed a similar pattern to that of England under the responsibility of Ministries of Health (formerly Ministries of Hygiene) or agencies similar in the national government, thus creating one of the most deceptive illusions concerning health: The illusion that mitigating, treating, caring and sometimes curing the disease employing an industrialized organization could improve the health of the population.

What we call health services are no more than medical services dedicated to the care of the disease - not even to those who suffer from them - ignoring health protection and promotion and a large part of preventing disease. Medicine has been used by big business to build profit-making industrial complexes that offer the consumer services of laboratories, diagnostic services, outpatient care and hospitalization. The use of increasingly sophisticated and expensive technologies leads to their concentration in diagnostic centers, clinics and hospitals, restricting access for a large segment of the population and constraining the historical practice of the medical professional in their consulting rooms^6^ ^-^ ^7^.

In this organization, they are no longer patients but change into what becomes customers, users or consumers subjected to two new risks: 1. clinical Iatrogenesis - when the patient-user-consumer (PUC) suffers complications, sequelae or death from services received; 2. social iatrogenesis - when people are subjected to medical power by means of medical certificates of health, fitness, disability, convalescence, etc., and become dependent on periodic medical visits and prescriptions for medications to "reduce risk" in healthy persons, or to adopt certain healthy lifestyles^8^.

All of this is supported by industrial power of so-called health services that push for medication for everyday life, creating a huge market for pharmaceuticals that are not always harmless. Probably, in the medium term the entire population that accesses these medical services will be daily taking curative, palliative, "preventive", and aesthetic medicines, or lifestyle modifiers. The medicalization of everyday life seems inexorable. Aesculapius is subjected to big business and Hygeia again begins to fade.

In 1974, Mark Lalonde in his famous report on the health of Canadians analyzed the fact that so-called health services are the personal medical services, not necessarily the most effective, and leaving out many interventions in the health field that produce much more health than can be done by medicine.

The contribution of these wrongly-called health services to reduce the risk of dying from various causes and what is spent in this reduction becomes such a waste that it should be fully disclosed to the population. In the USA, for example: To reduce mortality by 11% from all causes served by so-called health services 91% of total resources allocated to the health sector are spent; for reducing deaths from environmental causes by 19%, 1.5% of the total is spent; for reducing 27% of the biological causes of death 7% is spent, and for a 43% reduction in deaths from lifestyle causes barely 1.2% of the total resources allocated are spent^9^ ^-^ ^11^.

Some argue that health and disability insurance do not ensure the client's health but their pocketbook against the depredations of the medical system and reductions in earnings from disability for work^12^. Beveridge himself was very critical of the huge profits from health insurance and argued that "commercial interests should not be associated with the administration of the social welfare state"^13^.

The confusion with the so-called health services is such that in publications of the highest scientific prestige do not make distinctions with medical services, and worst blunders such as talking about preventive health and preventive health services are still committed (a complete incoherence), instead speaking of preventive medicine and preventive medical services. For example: "Education plays a role in people's attitudes about preventive health, as well as their expectations of health standards"^14^.

### True health services

Interventions that protect and promote health as well as help to prevent disease from occurring are the real health services. Therefore, we can call health services those services described in Table 1. True health services are provided by different organizations of the state and not only by just the so-called "health sector".

**Table 1:**
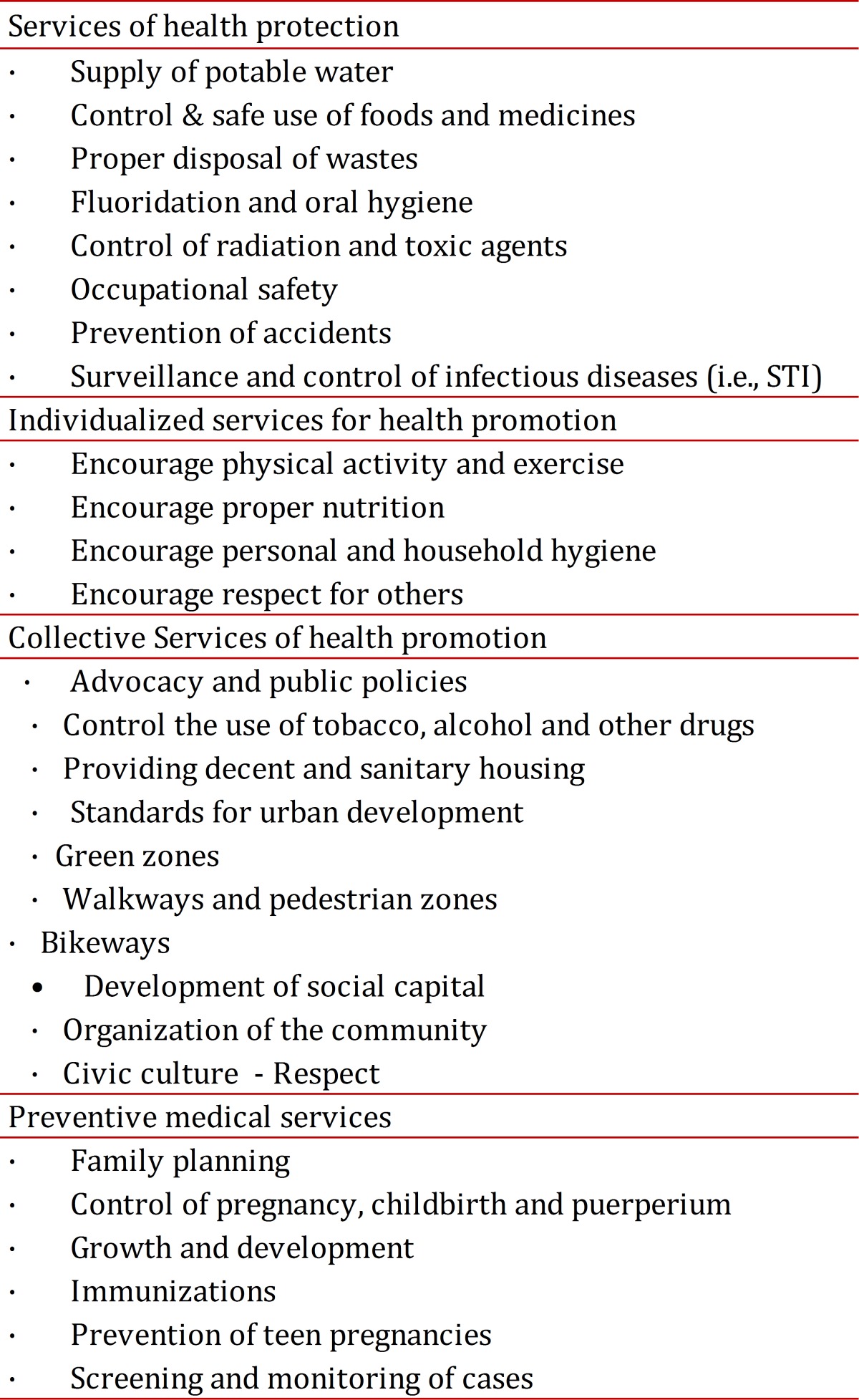
Health Services

Specifically, the services of health protection, such as the drinking water supply, proper disposal of waste and control of toxic agents and radiation are services that, in the case of the first two, have grown into big public businesses and, recently, into large private companies. Therefore they should remain among the most important health services that the state provides to the entire population.

Similarly, many of the services of health promotion are not directly supplied by what is called "the health sector". This only encourages and promotes precarious activities that modify the behavior and weakly try to influence the plans and programs in other sectors that enable healthy lifestyles.

The big challenge that true health services represent for the current "health sector" consists of radical reformation of the organization of the Ministry and health secretariats (today secretariats predominantly of medical services).

This reform must ensure the formation of sector alliances, capacity building to promote and encourage the organization of communities and strengthen the functions of leadership, advocacy and public policy analysis that are necessary to ensure the provision of true health services, regardless of the public or private institution that provides them.

To mimic medical services under the name of health services, society is deceived into believing that it urgently needs true health services as set out above and that are generally referred to under the generic name of public health. The true health services are those that have contributed preponderantly to improving health in the world^15^.

### Consequences

The importance of calling things by their right name is huge. Take the case of Colombia that merchandizes medicine: privatized medical services through a system of insurance paid for by employers, employees, and independent contractors, as well as by the government in the case of the poor population. Private intermediaries incorrectly called Health Promoting Companies (EPS), were created which are nothing more than for-profit insurers that in no way either protects or promotes the health of the population. Their power has grown to dominate the whole system for the provision of medical services, constraining the autonomy of medical practice, the freedom of patient choice and limiting benefits authorized by law.

In addition, the government allows them to recover copayments and prorated fees supposedly to accomplish "promotion and prevention," something they do not know how to do nor when to do it - it is a shameful pantomime of disease prevention and health promotion. Such a system of medical services has been enormously costly for all, highly inefficient, and in the end proletarianizes the medical profession. However, the medical associations continue to distantly think about individual benefits while the "system" surrenders to medical treatment operators and the patient becomes an unprotected and passive consumer.

Another very negative impact of so-called health care was the conversion of the Secretariats of Hygiene to Secretariats of Health that dedicate 80% of their effort to raising funds to guarantee health insurance for the poorest. Meanwhile, funds to control, reduce or eliminate priority health problems, such as the unacceptable outbreaks of dengue, the obesity epidemic, the spread of HIV/AIDS, the increase in new cases of tuberculosis and syphilis, among others, only reach levels up to 4% of the total national health budget^16^. Also, the organization of these secretariats is an example of the inconsistency between its true purpose and functions and the inefficiency in the actions that end up trying to guarantee the medical insurance imposed by the system.

Finally, it is worth noting that there is no intent here to be against medical services, but to be against the lie that disguises health care. In addition, the current form of financing for these services in the case of Colombia does not seem to be either more equitable or more efficient. An alternative that does seem to be more equitable and efficient is financing medical services with taxes or converting EPS into a non-profit organization. At the same time, true health services should be given all due importance by the State through adequate governmental budget appropriations for its efficient and effective operation. The University, for its part, should research true health services and train professionals to analyze public policies and advocate for ensuring equitable, efficient and effective health service delivery to the population. In the end, if one actually wants to improve the health of the population, it is necessary to vindicate Hygeia and make her a brother to Asclepius.
